# Emergence of carbapenem-producing enterobacteriaceae (CPE) and other multidrug-resistant gram-negative bacteria in neonates at a tertiary-level NICU in Tanzania: a point prevalence study

**DOI:** 10.1093/jacamr/dlaf179

**Published:** 2025-10-16

**Authors:** H Naburi, T Sewunet, C Tellapragada, N Nalitolela, M S Wranne, A Joachim, M Kasubi, M Mkony, F Westerlund, C G Giske, V Nordberg

**Affiliations:** Department of Paediatrics and Child Health, Muhimbili University of Allied Sciences, Dar es Salaam, Tanzania; Department of Paediatrics and Child Health, Muhimbili National Hospital, Dar es Salaam, Tanzania; Department of Laboratory Medicine, Karolinska Institutet, Stockholm, Sweden; Department of Clinical Microbiology, Karolinska University Hospital, Stockholm, Sweden; Department of Laboratory Medicine, Karolinska Institutet, Stockholm, Sweden; Department of Paediatrics and Child Health, Muhimbili University of Allied Sciences, Dar es Salaam, Tanzania; Department of Paediatrics and Child Health, Muhimbili National Hospital, Dar es Salaam, Tanzania; Department of Life Sciences, Chalmers University of Technology, Gothenburg, Sweden; Department of Microbiology and Immunology, Muhimbili University of Allied Sciences, Dar es Salaam, Tanzania; Department of Microbiology and Immunology, Muhimbili National Hospital, Dar es Salaam, Tanzania; Department of Microbiology and Immunology, Muhimbili University of Allied Sciences, Dar es Salaam, Tanzania; Department of Microbiology and Immunology, Muhimbili National Hospital, Dar es Salaam, Tanzania; Department of Paediatrics and Child Health, Muhimbili University of Allied Sciences, Dar es Salaam, Tanzania; Department of Paediatrics and Child Health, Muhimbili National Hospital, Dar es Salaam, Tanzania; Department of Life Sciences, Chalmers University of Technology, Gothenburg, Sweden; Centre for Antibiotic Resistance Research in Gothenburg (CARe), Gothenburg, Sweden; Department of Laboratory Medicine, Karolinska Institutet, Stockholm, Sweden; Department of Clinical Microbiology, Karolinska University Hospital, Stockholm, Sweden; Department of Clinical Science, Intervention and Technology, Karolinska Institutet, Stockholm, Sweden; Department of Neonatology, Karolinska University Hospital, Stockholm, Sweden

## Abstract

**Aim:**

Multidrug-resistant (MDR) Gram-negative bacilli pose a significant threat in neonatal care. This study aimed to evaluate the point prevalence and molecular characteristics of intestinal MDR colonization in neonates at Muhimbili National Hospital, Tanzania.

**Method:**

We conducted a point prevalence study with faecal samples from 51 neonates born ≥26 weeks gestational age (41% girls, mean 31.6 ± 3.8 weeks) admitted to the neonatal intensive care unit (NICU) at Muhimbili National Hospital on 17 May 2022. The median age at sampling was 8 days (interquartile range 11 days). Samples were cultured on chromogenic agar, and positive colonies underwent antimicrobial susceptibility testing. Whole-genome sequencing and plasmid analysis using Optical DNA Mapping (ODM) were performed on carbapenem-resistant isolates.

**Results:**

Among the 51 neonates, 31 (60.7%) were colonized by ESBL-producing *Klebsiella pneumoniae* (EP-KP) and/or *Escherichia coli* (EP-EC). Of these, 15 isolates were carbapenem-producing Enterobacteriaceae (CPE) harboring *bla*_NDM-5_, *bla*_CTX-M-15_, and eight also carried *bla*_OXA-181_. The most prevalent carbapenemase-producing *Klebsiella pneumoniae* (CP-KP) sequence type (ST) was ST437, part of the high-risk clonal complex CC11, while the most common carbapenemase-producing *E. coli* (CP-EC) was ST167. Both CP-KP and CP-EC were MDR isolates encoding *bla*_CTX-M-15_ and *bla*_NDM-5_. Optical DNA Mapping showed that the *bla*_NDM-5_ encoding plasmids in at least six carbapenem-producing isolates (four KP ST437 and two EC ST167) were similar, suggesting plasmid transfer.

**Conclusion:**

A high prevalence of colonization with high-risk clones was observed in neonates, highlighting the urgent need for strengthened MDR-surveillance, infection control, and antibiotic stewardship in the NICU at MNH.

## Background

Each year, approximately one million newborns die from neonatal sepsis, with antimicrobial-resistant bacteria contributing to one-third of these deaths.^1–3^ Antimicrobial resistance (AMR) is a growing global health threat, with projections indicating that by 2050, an AMR-related death will occur every 3 s if current trends persist.^4^ Consequently, the World Health Organization (WHO) has made AMR its top priority, aiming to reduce mortality rates from bacterial infections.^5^ To tackle this issue, the WHO AWaRe classification of antimicrobials recommends the appropriate use of antibiotics to reduce the development and spread of AMR.^6^ A major challenge in AMR is the emergence of multidrug-resistant (MDR) strains, such as *Escherichia coli* (EC) and *Klebsiella pneumoniae* (KP), which often carry genes for extended spectrum beta-lactamase (ESBL) and carbapenemase production.^7^ The presence of ESBL-producing Enterobacteriaceae (EPE) and carbapenemase-producing Enterobacteriaceae (CPE) in the neonatal intestinal microbiota can precede clinical infections, such as necrotizing enterocolitis, bloodstream infections, and urinary tract infections.^8,9^ In Eastern Africa, the prevalence of EPE is high, with up to 50% of hospital isolates, mainly EC and KP.^10^ The Global Health Security Agenda report identifies AMR as a major issue in Tanzania.^11^ Regional studies indicate varying degrees of resistance, with high EPE prevalence in Dar es Salaam and northern Tanzania, and notable levels of CPE in Mwanza.^12–15^

Risk factors for neonatal colonization with EPE include prematurity, maternal intestinal colonization, prolonged hospitalization, and prior antibiotic exposure.^16^ Patterns of early EPE-colonization vary by setting: vaginal delivery is often associated with increased risk in high-income countries, whereas hospital-related factors predominate in low- and middle-income regions. A study conducted in Tanzanian reported high rates of EPE-colonization in neonates and their environment, with hospital admission and cesarean delivery elevating risk, whereas vaginal delivery was protective.^17^ This colonization increases the risk of transmission, difficult-to-treat infections and higher neonatal mortality. Understanding local resistance patterns in neonates is crucial for selecting appropriate treatments and preventing the spread of resistant strains.^18^

This study investigated the burden and molecular characteristics of MDR Enterobacteriaceae colonizing neonates at the neonatal intensive care unit (NICU) of Muhimbili National Hospital (MNH), Tanzania. Specifically, we aimed to determine the prevalence of intestinal colonization with EPE and CPE in hospitalized neonates, identify the associated resistance genes, and explore potential plasmid transfer between EC and KP isolates using Optical DNA Mapping (ODM). We further assessed antibiotic use in the NICU in relation to the WHO AWaRe classification system, with the goal of contributing data to guide future infection control strategies and antimicrobial stewardship at MNH.

## Methods

### Ethics

Ethical approval was obtained from the Institutional Review Board of the Muhimbili University of Health and Allied Sciences (MUHAS-REC-07-2021-770) and the National Institute for Medical Research (NIMR/HQ/R.8a/Vol. IX/3660). Permission to conduct the study was granted by the Research, Training, and Consultancy Unit hospitals (MNH/TRCU/Perm/2021/078). This research was carried out in line with the Declaration of Helsinki and complied with all relevant national and institutional ethical regulations. Participation was voluntary, and written informed consent was obtained from all mothers prior to data collection.

### Study description, settings, study population

This study was a one-day point-prevalence investigation conducted on 17 May 2022. Rectal swabs were obtained from 51 inborn neonates, all born at or after 26 weeks of gestational age, admitted to the NICU at MNH. All neonates admitted to the NICU whose mothers provided both written and verbal informed consent were included in the study. Neonates with anorectal malformation were excluded. Due to overcrowding in the NICU, cot-sharing of infants occurred. However, all data collection and sampling took place on a single day, and no infants were moved between cots during this period.

MNH is the largest tertiary care hospital in Dar es Salaam, Tanzania. The hospital serves as a referral centre from regional hospitals both within and outside the Dar es Salaam region and functions as a key teaching hospital. The NICU at MNH has 100 beds and admits ∼4000–4500 neonates annually, with about 120–150 at any given time. The unit provides level III NICU services. The hospital’s central pathology laboratory (CPL) conducts various tests, including blood cultures, antimicrobial susceptibility testing, and clinical biomarkers for sepsis. First-line empirical therapy for early onset neonatal sepsis (EOS) at MNH follows the WHO guidelines, using ampicillin/cloxacillin combined with an aminoglycoside, such as gentamicin. Second-line therapy for EOS and first line treatment for late onset sepsis (LOS) includes a third-generation cephalosporin (cefotaxime or ceftriaxone) or ciprofloxacin. For third-line treatment, meropenem or piperacillin-tazobactam (PTZ) is used. Antibiotic choices for both EOS and LOS are intended to be adjusted based on culture and susceptibility testing results, according to guidelines.

### Bacterial culturing of faecal samples

Rectal swabs were collected using sterile COPAN transport tubes (Copan Diagnostics, CA, USA) and transported to the CPL at MNH. The swabs were inoculated on CHROMagar^™^ (bioMérieux, Marcy l’Etoile, France) and incubated at 37°C for 18–24 h. Bacterial identification was performed using biochemical tests (indole and citrate test) and the Analytical Profile Index 20E and confirmation of ESBL-production using Combination disk testing method at CPL. All ESBL-positive isolates, and additionally those with phenotypical carbapenem-resistance were preserved in nutrient broth containing 15% glycerol at −80°C and shipped on dry ice to Karolinska Institutet, Stockholm, Sweden, for molecular characterization and extended antimicrobial susceptibility testing.

### Bacterial identification and antimicrobial susceptibility testing

Species identification of isolates at the Karolinska Institutet laboratory was performed by MALDI-TOF (Bruker Daltonics, MA, USA). ESBL production was confirmed by the combination disk testing (CDT) method (Oxoid, Basingstoke, UK). Antimicrobial susceptibility testing by disk diffusion was determined for amikacin (AK), ciprofloxacin, ertapenem, gentamicin, imipenem, meropenem, PTZ and trimethoprim-sulfamethoxazole (Oxoid). Zone diameters were interpreted according to the EUCAST clinical breakpoints (v13.1). Isolates with zone diameters <28 mm against meropenem were identified as putative carbapenemase producers and further subjected to whole-genome sequencing for confirmation.^19^

### DNA extraction and WGS

Genomic DNA was extracted from freshly cultured colonies of carbapenem-resistant EC and KP isolates using the EZ1 DNA Tissue kit (Qiagen, Hilden Germany) on an automated DNA extraction system, EZ1Advanced XL system (Qiagen, Hilden, Germany). The extracted DNA was quantified using the Qubit DS DNA-High Sensitivity Kit (Thermo Fisher, Waltham, USA). Sequencing libraries were prepared using the Nextera XT Kit (Illumina, San Diego, CA, USA) and subjected to paired-end short-read sequencing Illumina (NovaSeq 6000, Illumina, San Diego,CA,USA) at the Science for Life Laboratory, Stockholm, Sweden. Quality control and assembly of the raw sequences were performed using FastQC (v 0.11.8) and SPAdes (v 3.15.5). Multi-locus sequence typing (MLST) of the isolates was performed using the public database (https://cge.food.dtu.dk/services/MLST/) and AMR genes were detected using ResFinder.

### Plasmid extraction and ODM

Plasmids were extracted using the NucleoBond^®^ Xtra Midi kit (Macherey-Nagel) according to the manufacturer’s instructions. The final DNA concentration was measured using Qubit^™^ dsDNA BR Assay Kit (ThermoFisher). Plasmids from six of the 15 carbapenem-resistant isolates were analyzed using ODM in combination with CRISPR/Cas9.^20^ Cas9, along with guide RNA (gRNA), was used to identify plasmids encoding either *bla*_NDM-5_ or *bla*_CTX-M-15_. In brief, plasmids were first mixed with Cas9 to linearize those encoding the gene of interest (*bla*_NMD-5_ or *bla*_CTX-M-15_). The plasmids were then stained with YOYO-1 and netropsin. This staining process creates sequence-dependent intensity variations along the DNA as the non-fluorescent netropsin blocks YOYO-1 from binding to AT-rich regions, creating an emission intensity variation along the DNA that corresponds to the underlying sequence, referred to as a ‘DNA barcode’. The DNA was then stretched using a nanofluidic device and imaged with an inverted epi-fluorescence microscope.^21^ In an inverted epi-fluorescence microscope the fluorescence from the sample is studied from below (in this case the bottom of the nanofluidic device) with both excitation and emission light travelling through the same objective, which is positioned below the sample instead of above. The images were analyzed using a set of custom-made MATLAB scripts.^20^ First, the intensity traces of each DNA molecule in the images were extracted. If a plasmid has been cut by Cas9 there will be several DNA molecules with the same intensity pattern and cut site. From this data, a consensus barcode was extracted. The consensus barcodes of plasmids from different isolates were then compared with each other to assess if the same plasmid was present in several isolates. A *P*-value was used to assess the similarity; if the *P*-value was <0.001 the plasmids were considered similar.^22^

### Statistical analysis

Data were exported from Excel database to the Stata version 18 [Stata Corporation; College Station, USA] for analysis. The characteristics of the neonates screened were summarized as percentages for categorical variables, and as means and standard deviations or medians and interquartile ranges (IQR), as appropriate for continuous variables. Due to the relatively small sample size and study design, analyses were limited to descriptive statistics without inferential testing.

## Results

### Clinical characteristics of the study population

The characteristics of all neonates screened are presented in Table 1. The screened group consisted of 51 neonates, of which 21 (41%) were female. The median age at sampling was 8 days, with an IQR of 11 days. Sixteen (31.3%) of the neonates exhibited documented clinical signs of sepsis, but no blood culture results were available to confirm a bacterial aetiology. Eighteen (35.5%) neonates had documented antibiotic use prior to or at the time of screening. The median duration of antibiotic use was 6.5 days (IQR 5). Due to missing data, antibiotic selection was documented for only 14 neonates. Of these, 11/14 (78.6%) were prescribed an Access antibiotic, 3/14 (21.4%) a Watch antibiotic, and none on a Reserve antibiotic. Access antibiotics included ampicillin and gentamicin, whereas cefotaxime, ceftriaxone and ciprofloxacin were categorized as Watch antibiotics. At the time of screening, seven (23.3%) of the 30 neonates with documented information had been transferred within the ward, and nine (30%) had had their cots changed.

**Table 1.** Characteristics of the screened neonates

Variables	*N* (%)
Gestational age (weeks) mean (SD)	31.6 (3.8)
<28	8 (15.7)
28–<32	15 (29.4)
32–<34	8 (15.7)
34–<37	17 (33.3)
≥37	3 (5.9)
Birth weight (grams) mean (SD)	1584 (485)
<1000	5 (9.8)
1000–<1500	8 (15.7)
1500 < 2500	24 (47.1)
≥2500	14 (27.4)
Colonized with EPE	*n* = 51
Yes	32 (62.8)
No	19 (37.2)
Age in days at sampling median (IQR)	8 (11.0)
Mode of delivery	*n* = 37
Spontaneous vaginal delivery	19 (51.4)
Caesarean section	18 (48.6)
Apgar at 5 min: mean (SD)	8 (1.6)
Sex	*n* = 39
Male	18 (46.2)
Female	21 (53.8)
Place of birth	*n* = 38
Home	3 (7.9)
Hospital	29 (76.3)
Health centre	6 (15.8)
Required Resuscitation	*n* = 40
Yes	21 (52.5)
No	19 (47.5)
Feeding method	*n* = 35
Breastfeeding	6 (17.1)
Cup feeding	10 (28.6)
Tube feeding	19 (54.3)
Duration of hospital stay in days; Median (IQR)	7 (11.0)
Clinical signs of sepsis documented	*n* = 30
Yes	16 (53.3)
No	14 (46.7)
On antibiotics before or during screening	*n* = 21
Yes	17 (81.0)
No	4 (19.0)
Duration of antibiotics in days: median (IQR)	6.5 (5.0)
Antibiotic groups	14
Access (ampicillin, gentamicin)	11(78.6)
Watch (cefotaxime, ceftriaxone, ciprofloxacin)	3 (21.4)
Reserve	0 (0)
Transfer within ward before EPE-screening	*n* = 30
Yes	7 (23.3)
No	23 (76.7)
Cot changed during stay	*n* = 30
Yes	9 (30)
No	20 (70)

Number for each variable varies as some of patient characteristics were missing.

EPE, ESBL-producing *Enterobacteriaceae*; IQR, Interquartile range; SD, standard deviation.

### Microbiology results

In total, 41 EPE isolates (19 EP-EC isolates, 21 EP-KP isolates and one EP-*K. oxytoca* (KO) isolate were isolated from 31/51 (60.7%) of the neonates screened. One EPE was isolated from 21 neonates and two EPE in 10 neonates. Resistance to ceftriaxone was observed in 100% of the EPE-isolates. The highest resistance was observed against trimethoprim-sulfamethoxazole (84% in EC and 95% in KP) followed by ciprofloxacin (63% in EC and 71% in KP). Table 2 presents the proportion of isolates exhibiting resistance to each of the antibiotics tested. Based on the susceptibility results, 15 EPE isolates from 14 samples were presumptively identified as resistant to carbapenem (meropenem zone diameter < 28 mm). These 15 isolates, comprising 10 KP, 4 EC and 1 KO were subjected to whole-genome sequencing. The remaining isolates were confirmed using phenotypic methods. All the 15 carbapenem-resistant isolates carried both *bla*_NDM-5_ and *bla*_CTX-M-15_, while eight of the isolates also harbored the *OXA-181* gene. Eight carbapenemase producing KP (*bla*_NDM-5_ and *bla*_OXA-181_) belonged to ST437 and the remaining two KP belonged to ST392 and ST147, respectively. All four EC isolates were classified as ST167. One *Klebsiella oxytoca* isolate carried *bla*_NDM-5_ and *bla*_CTX-M-15_ genes and lacked an assigned ST.

**Table 2.** Proportion of isolates with resistance to individual antibiotics tested

Isolate	PTZ *n* (%)	MEM *n* (%)	ETP *n* (%)	IMI *n* (%)	AK *n* (%)	GEN *n* (%)	SXT *n* (%)	CIP *n* (%)
EC (*n* = 19)	9 (47)	6 (31)	6 (31)	5 (26)	5 (26)	9 (47)	16 (84)	12 (63)
KP (*n* = 21)	13 (62)	8 (38)	9 (43)	9 (43)	5 (24)	12 (57)	20 (95)	15(71)

AK, amikacin; CIP, ciprofloxacin; EC, *E. coli*; ETP, ertapenem; GEN, gentamicin; IMI, imipenem; KP, *K. pneumoniae*; MEM, meropenem; PTZ, piperacillin-tazobactam; SXT, trimethoprim-sulfamethoxazole.

### ODM

**Figure 1.**
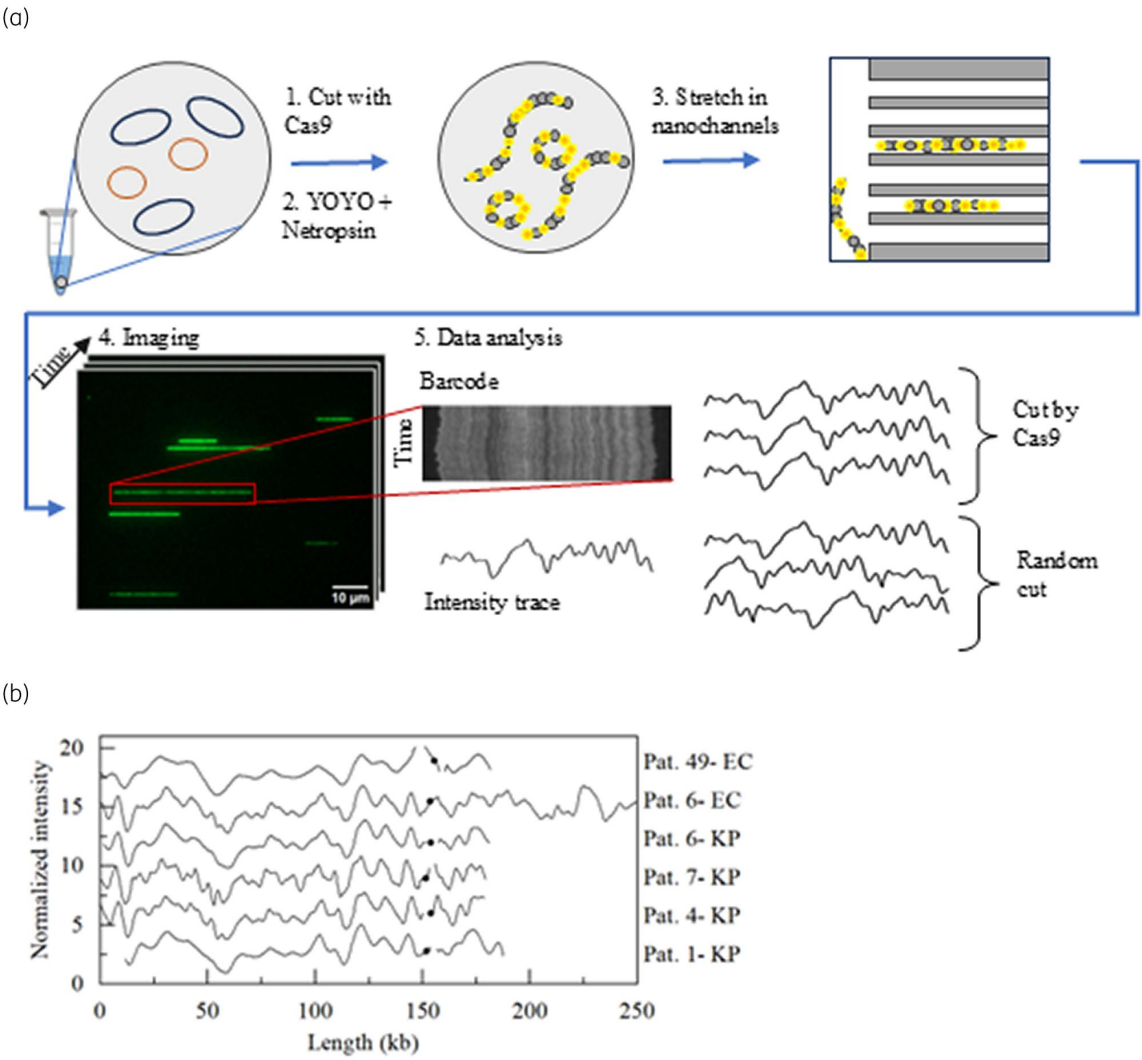
(a) CRISPR/Cas9 and ODM on resistant bacterial isolates. (b) Plasmids with blaNDM-5; black dot indicates location of blaNDM-5.

Next, we investigated whether the same plasmid was present in EC ST167 and KP 437 strains, which would suggest plasmid transmission. To explore the potential use of ODM as a method for assessing clonal relatedness or identifying local outbreak in low-resource settings, a subset of isolates was selected based on feasibility. Plasmids from six isolates four KP ST437 and two EC ST167 were analyzed using ODM (Figure 1). The six samples contained 3–4 plasmids each, with sizes from 58 to 211 kb. We used Cas9 to target *bla*_NDM-5_ or *bla*_CTX-M-15_. To identify the plasmids carrying the resistance genes. In all six isolates one plasmid encoding *bla*_NDM-5_ was identified, in five cases that plasmid was around 150 kb (151.4 ± 2.8 kb) and in one case (Pat 6-EC) the plasmid was significantly longer, 211 kb. Despite the length difference the six plasmids encoding *bla*_NDM-5_ all showed high similarity (*P*-value < 0.001). The (Pat 6-EC) sample have an insert of around 60 kb that is not present in the other plasmids, but is otherwise very similar to the other plasmids. The location of the *bla*_NDM-5_ gene was also very similar between samples. *bla*_CTX-M-15_ was not found in the plasmids in any of the investigated isolates, indicating that the *bla*_CTX-M-15_ gene might be encoded on the chromosome.

## Discussion

The prevalence of neonates colonized by EPE and CPE was high. Our findings are consistent with three previous studies conducted in neonatal wards in Tanzania that reported high prevalence of EPE and occurrences of invasive infections involving EP-EC and EP-KP.^23–25^ Several studies have demonstrated a strong association between sepsis caused by EPE-strains and increased mortality, with a substantial proportion attributed to KP.^24,26^

It is clinically concerning that Gram-negative bacteria causing neonatal sepsis have become increasingly resistant to aminoglycosides and third-generation cephalosporins, which are recommended by the WHO for empirical treatment.^27,28^ At MNH, empirical broad-spectrum antibiotics are initiated when sepsis is suspected, with escalation to more targeted or broader spectrum agents if there is no improvement while awaiting blood culture results. Delays in blood culture turnaround times often result in prolonged antibiotic use. As a tertiary referral hospital, MNH receives critically ill neonates and high-risk pregnant women, some of whom may have already received multiple courses of antibiotics, limiting empirical treatment options. Due to missing data, we were unable to fully assess antibiotic use according to the AWaRe guidelines. However, 78.6% of neonates received Access antibiotics, 21.4% received Watch antibiotics, and none received Reserve antibiotics. These proportions were different than national data (2020–2022), which reported 53.6% Access use (a 13% decrease from 2020), 25.6% Watch use (a 6% increase), and no recorded Reserve use.^29^ As no neonates had a positive blood culture and antibiotics were administered empirically in the absence of culture-confirmed sepsis, assessing the appropriateness of treatment was challenging. The KP ST437 isolates identified in this study are part of a high-risk clonal complex (CC11) that is widely distributed across multiple countries. Historically, isolates belonging to ST437 were predominantly *bla*_KPC-2_ and *bla*_CTX-M-15_ producers and known to cause outbreaks. Few recent studies in Europe have reported ST437 strains producing *bla*_NDM_ and/or *bla*_OXA-48_ enzymes.^27,28^ KP ST437 isolates producing *bla*_NDM-5_, as observed in this study, are relatively rare and have so far been reported from Italy and India.^30^ The EC ST167 harboring *bla*_NDM-5_ is an internationally recognized high-risk clone,^31^ known for its extensive drug resistance and first identified in a Tanzanian neonate in 2018.^32^ The four EC ST167 isolates identified in this study were resistant to all the antibiotics tested except AK. In a recent study with environmental samples from the paediatric wards at MNH, 22% of the Gram-negative samples tested positive for CPE using the phenotypic method, modified carbapenem inactivation method. No sequencing was performed in that study.^33^

A novel aspect of our study was the use of ODM to characterize plasmids, allowing identification of whether the same *bla*_NDM-5_ encoding plasmid was present in different patients and different bacterial species. The same plasmid was found in several EP-KP and EP-EC isolates, suggesting potential horizontal transfer both between species and among neonates in the NICU. This underscores the significance of plasmid-mediated resistance transmission in this vulnerable population. Overcrowding and understaffing, common in LMICs, exacerbate the challenges of implementing outbreak surveillance and infection control measures.^34^ In settings with endemic levels of MDR, the transmission dynamics are heavily influenced by environmental factors within the neonatal ICU.^35^

Implementation of EPE-screening to predict MDR-bacterial infections in neonates has been adopted in several countries, as an important tool for hygiene and MDR-surveillance, although its usefulness for guiding empirical treatment in suspected neonatal infections remains limited.^36^ MNH has not yet implemented this screening routine.

This study has some important limitations. The point-prevalence design and relatively small sample size (51 neonates) limit generalizability and prevent assessment of trends or causality. Furthermore, while some risk factors- such as maternal antibiotic use and AWaRe antibiotic groups- were descriptively reported (Table 1), the study was not powered to perform a formal risk factor analysis. Analyses were limited to descriptive statistics without inferential testing due to the small sample size and study design. Given that key risk factors for neonatal ESBL colonization are well established in the literature, our primary aim was to documentthe point prevalence and magnitudethe problem in this setting to inform IPC measures and guide antimicrobial stewardship. Future studies should consider longitudinal designs and multicenter neonatal cohorts to better understand colonization dynamics and improve external validity. Additionally, the lack of individual-level data on cot sharing- an important factor in colonization dynamics- represents another limitation and should be considered in future research.

### Conclusions

This study found that the majority of neonates at MNH were colonized with EPE, with a substantial proportion exhibiting carbapenem resistance. Most neonates received Access antibiotics, however missing data precluded a complete AWaRe classification analysis. High-risk clones producing *bla*_NDM-5_ were detected, suggesting potential horizontal transmission in the NICU. These findings underscore the need for improved infection control, rapid diagnostics, and antibiotic stewardship in neonatal care. This study provides an important first step towards addressing these challenges and enhancing the intervention effectiveness in the NICU at MNH.
